# Hepatitis E virus as a Cause of Acute Hepatitis in The Netherlands

**DOI:** 10.1371/journal.pone.0146906

**Published:** 2016-02-03

**Authors:** Aletta T. R. Tholen, Janke Schinkel, Richard Molenkamp, C. Wim Ang

**Affiliations:** 1 Department of Medical Microbiology and Infection Control, VU University Medical Center, Amsterdam, The Netherlands; 2 Section of Clinical Virology, Department of Medical Microbiology, Academic Medical Center, University of Amsterdam, Amsterdam, The Netherlands; University of Cincinnati College of Medicine, UNITED STATES

## Abstract

**Background:**

Recent studies indicate that 27% of Dutch blood donors have evidence of past infection with HEV. However, the low number of diagnosed HEV infections indicates either an asymptomatic course or under diagnosis.

**Objectives:**

We investigated whether HEV is a cause of acute hepatitis in Dutch patients and which diagnostic modality (serology or PCR) should be used for optimal detection.

**Study design:**

Serum samples were retrospectively selected from non-severely immuno-compromised patients from a university hospital population, suspected of having an infectious hepatitis. Criteria were: elevated alanine aminotransferase (ALT> 34 U/l) and request for antibody testing for CMV, EBV or Hepatitis A (HAV).

**Results:**

All samples were tested for HEV using ELISA and PCR. Ninety patients/sera were tested, of which 22% were HEV IgG positive. Only one serum was IgM positive. HEV PCR was positive in two patients: one patient was both HEV IgM and IgG positive, the other patient was only IgG positive. Both HEV RNA positive samples belonged to genotype 3. Evidence of recent infection with CMV, EBV and HAV was found in 13%, 10% and 3% respectively.

**Conclusions:**

Although our study is limited by small numbers, we conclude that HEV is a cause of acute hepatitis in hospital associated patients in The Netherlands. Moreover, in our study population the prevalence of acute HAV (3%) was almost similar to acute HEV (2%). We propose to incorporate HEV testing in panels for acute infectious hepatitis. Negative results obtained for HEV IgM in a HEV PCR positive patient, indicates that antibody testing alone may not be sufficient and argues for PCR as a primary diagnostic tool in hospital associated patients. The high percentage of HEV IgG seropositivity confirms earlier epidemiological studies.

## Background

Hepatitis E (HEV) is an important cause of acute hepatitis and jaundice worldwide [1]. Several lines of evidence suggest that HEV is more frequent in non-endemic regions than was suspected. Recent seroprevalence studies with assays that detect long-lasting IgG responses showed seroprevalence rates of ranging from below 5 to over 20% in blood donors in developed countries [2]. Genotype 3 is the most prevalent HEV genotype in The Netherlands and other industrialized countries [3, 4].

The epidemiology of HEV in the Netherlands has been studied extensively. The HEV seroprevalence pattern in Dutch blood donors from several periods between 1988 and 2011 suggests that several decades ago, HEV was ubiquitous in the Netherlands and a large proportion of the population became HEV infected [5]. Subsequently HEV incidence was low during a prolonged period to increase significantly again in recent years [6]. Most of the HEV infections in the Netherlands have been acquired in the Netherlands itself and only a minority is travel-associated [3].

The contribution of HEV in patients presenting with acute hepatitis in developed countries is not completely clear. Different studies reported evidence for HEV infection in 2–11% of patients [4, 7–13]. However, the estimated prevalences are not comparable, since the chosen diagnostic modalities and the study populations in these studies are different. In some studies, patients who tested positive for CMV, EBV, Hepatitis A (HAV), B (HBV) or C (HCV) were excluded [4, 7–10, 12, 14]. In other studies, serological testing only occurred in a selected group of patients suspected for having HEV, or the study population was not well defined [9, 11]. Finally the diagnostic methods differed widely, including different ELISA’s, immunoblot and PCR. PCR is preferred above serology in severely immunocompromised patients, since serology can be false-negative [15]. In immunocompetent patients, serology alone is considered to be sufficient [16]. However the optimal diagnostic strategy to diagnose HEV in patients who have comorbid conditions that will impair their immune system but are not severely immunocompromised remains unclear.

The aim of this study was therefore to investigate the frequency of HEV as a cause of infectious hepatitis in non-severely immunocompromised patients in a typical hospital population, and which diagnostic modality, IgG/IgM ELISA versus PCR, should be used to diagnose a HEV infection.

## Study Design

From November 1^st^, 2011 until October 31^st^, 2012, 90 consecutive serum samples from patients that were suspected of having an infectious hepatitis were selected at the VU Medical Centre in Amsterdam, the Netherlands, a tertiary care university hospital. Selection criteria were: elevated alanine aminotransferase (ALT> 34 U/l) and a request for antibody testing for CMV, EBV, HAV or HEV within 3 weeks of ALT testing. Because we wanted to investigate non-severely immunocompromised patients, we excluded patients with a severe immunocompromised state (e.g. oncology patients during chemotherapy, solid organ or bone marrow transplant patients). Other exclusion criteria were age less than 17 years old, chronically elevated ALT or any non-infectious cause of an increased ALT (e.g. cholecystolithiasis or liver metastasis). A concurrent acute or chronic HBV, HCV or HIV infection was not an exclusion criterium.

All samples were tested for anti-HEV immunoglobulin IgG and IgM antibodies by ELISA (Wantai Pharmaceutical Co., Beijing, China). IgM and IgG antibodies against CMV and HAV were tested with Architect (Abbott, Lake Forest, Illnois) and against EBV (IgM VCA, IgG VCA, IgG EBNA) with Liaison (Diasorin, Saluggia, Italy). Samples were screened for HEV RNA by using real-time reverse transcription PCR (RT-PCR) with primers detecting all 4 genotypes and validated according to International Standards Organization guidelines 9001 and 15189 [15]. The HEV ORF3 genomic region (nt 5292–5369, D11093) is highly conserved based on the alignment of full-length HEV sequences from various genotypes isolated from humans and swine [17]. The primers for the realtime fluorescent RT-PCR were: sense primer: 50-CGGTGGTTTCTGGGGTGA-30 and antisense primer: 50- GCR AAG GGR TTG GTT GG -30 (Acc No.D11093, nt 5261–5367). The probe was: FAM- 5’- ATT CTC AGC CCT TCG C- 3’MGB-NFQ. The PCR reaches a sensitivity of 250 IU/ml according to the probit analysis. To assess the origin of the virus involved, HEV genotyping was performed as described previously [18].

Because the samples were anonymized and recoded no consent was needed, according to local VU University Medical Center and national legislation. The study was approved by the scientific committee of the department of Medical Microbiology and Infection prevention of the VU University Medical Center, according to VU University Medical Center regulations.

## Results

The study population consisted of 45 males and 45 females with an age range from 17–81 years (mean 42.1). By definition, all patients had an elevated ALT (mean 258 U/L). Other tests for liver function were also abnormal (AST mean 155, gamma-GT mean 189, alkaline phosphatase mean 149). Total bilirubin was only elevated in a subgroup of patients and albumin levels were mostly within normal range. A prothrombin test was performed in only one patient with a result within normal range. Twenty out of 90 (22%) serum samples were HEV IgG positive. Only one sample was IgM positive.

The HEV PCR was performed once on all samples and positive in two patients. One patient had a positive HEV IgM- and IgG- response, the other patient was HEV IgM negative and IgG positive. Both HEV RNA positive samples were amplified on the ORF 1 and 2 region and belonged to genotype 3.

The patient with HEV IgM negative/PCR positive test results was a 36-year-old pregnant woman originally from Portugal with a medical history of pre-eclampsia and partus prematurus. She presented with fever, muscle pain and an increased ALT of 108 IU/L. The diagnosis was made retrospectively. An earlier sample of this woman, taken three months earlier, was HEV IgM/IgG negative, no HEV RNA testing was performed because we did not have enough material for a reliable HEV PCR. She made a complete recovery and gave birth to a healthy child. The child was not tested for HEV. The other patient, IgM and PCR positive, was a 70-year-old male. He had a medical history of type 2 diabetes mellitus, cardiovascular disease and alcoholic pancreatitis. His maximum ALT was 182 IU/L.

Evidence of recent infection with CMV, EBV and HAV, defined as being IgM positive, is shown in Table 1. We did not observe patients infected with multiple viruses.

**Table 1:** Results of viral testing from 90 patients suspected of having an infectious hepatitis.

Pathogen	Acute infection* (%)	PCR positive (%)	IgM/IgG positive (%)	IgM negative/ IgG positive (%)
**CMV**	12 (13%)			
**EBV**	9 (10%)			
**Hepatitis A**	3 (3%)			
**Hepatitis E**	2 (2%)	2 (2%)	1 (1%)	20 (22%)

* Acute infection is defined as IgM and/or PCR positivity for tested pathogen

## Discussion

In this study we found evidence for a recent infection with HEV in 2% of patients suspected for infectious hepatitis which is comparable to HAV (3%). CMV and EBV accounted for more cases and together these four viruses were responsible for 40% of all episodes of acute hepatitis cases in our study population.

We aimed at investigating a typical hospital population in an industrialized country suspected for viral hepatitis. We have labeled this group with the adjective “non-severely immunocompromised”. We excluded severely immunocompromised patients because this patient group has been studied previously and it is already known that PCR is indispensable for diagnosis because the serological response is often delayed or absent [15]. For immunocompetent patients, anti-HEV IgM and IgG detection are considered to be sufficiently sensitive for diagnosing acute HEV infection, including genotype 3 [16, 19]. However, the current hospital population, both inpatient and outpatients, includes many patients that either have a disease or use drugs that will affect their immune system. We tried to define this group of patients by excluding patients with severe immunosuppression. The two patients that were diagnosed with HEV, both had a relative immunosuppressed state (one pregnant woman, one alcoholic diabetic) and the pregnant woman did not have a detectable IgM response, indicating that for a hospital population, antibody testing may not be sufficient. The discrepancy between antibody testing and PCR has been observed in earlier studies [8, 19, 20], but for the non-severely immunosuppressed patients, no clinical details were available. Echevarria et al. tested 158 patients with both HEV RNA and a HEV IgM/IgG recombinant immunoblot from Mikrogen and observed that out of 15 sera tested positive for HEV RNA, three were negative for anti-HEV IgM and one indeterminate [8]. Moreover, out of 18 serum samples tested positive for anti-HEV IgM, seven serum samples appeared negative with PCR [8]. In the study of Pas et al, the diagnostic performance of eight serological tests, including the Wantai assay used in this study, were compared to HEV PCR [19]. In 88 HEV PCR confirmed cases the IgM sensitivity ranged from 52 to 81% [19]. El-Sayed Zaki et al. used a nested PCR to detect HEV RNA in Egyptian children and found that only 4/15 HEV PCR positive children had an IgM response as measured with a Genelabs assay [20].

Previous studies that have investigated the HEV as a cause of infectious hepatitis were mainly based on the analysis of unselected laboratory records or ill-defined populations [4, 8, 12, 21]. The major drawback of this approach is a selection bias towards more severe cases. We circumvented this selection bias by including all patients that were suspected for an infectious hepatitis. Patients with a request for HAV infection were not excluded so we could compare the relative contribution of HAV to HEV. Almost no prevalence data about HAV compared to HEV are available. Two recent laboratory based studies in the UK reported a higher frequency of acute HEV infection than that of HAV. In both studies, not all patients were tested for both HEV and HAV antibodies and no clear selection criteria for HEV testing were defined [4, 21].

Although most infections with HEV are relatively mild, HEV was the cause of acute liver failure in 10–15% of patients [22]. In our patient group none of the HEV patients met the criteria of acute liver failure.

As we studied patients from both outpatient clinics and hospitalized patients, we cannot extrapolate our results to a general population or a GP population. An earlier study from the Netherlands indicates that the fraction of HEV infections in the general population may even be higher than reported in our study [9].

The choice of antibody assay can strongly influence the results. Recently, a comparative study of HEV ELISA’s was published. The authors found a diagnostic sensitivity for IgM of 74% with the Wantai ELISA that was used in this study, that could explain our false-negative IgM sample [19]. In our study-population, HEV PCR as a sole method would have been sufficient to detect all cases but in other studies PCR-negative, IgM positive cases have been described [8, 20].

Our observation that 20% of patients were HEV IgG seropositive confirms earlier epidemiological studies [18]. A single positive result for HEV IgG is therefore not conclusive for an acute HEV infection in countries/regions with high IgG seroprevalence.

We can conclude that HEV is a relevant cause of acute hepatitis in The Netherlands. We propose that HEV testing is included in viral hepatitis panels. The optimal strategy for diagnosing HEV in a hospital setting may include both serology and PCR.
